# A sustained high fat diet for two years decreases IgM and IL-1 beta in ageing Wistar rats

**DOI:** 10.1186/s12979-015-0040-1

**Published:** 2015-09-28

**Authors:** Georg Pongratz, Torsten Lowin, Robert Kob, Roland Buettner, Thomas Bertsch, L. Cornelius Bollheimer

**Affiliations:** Department of Rheumatology, Hiller Research Center Rheumatology, University of Duesseldorf, Moorenstr. 5, 40225 Duesseldorf, Germany; Department of Internal Medicine I, University of Regensburg, 93042 Regensburg, Germany; Institute for Biomedicine of Ageing, Friedrich-Alexander-Universität Erlangen-Nürnberg, Nuremberg, Germany; Department of Internal Medicine, Klinik Bogen, 94327 Bogen, Germany; Institute of Clinical Chemistry, Laboratory Medicine and Transfusion Medicine, Paracelsus Medical University, Nuremberg, Germany; Department of General Internal Medicine and Geriatrics, Hospital of the order of St. John of God, Regensburg, Germany

**Keywords:** B cell function, High-fat diet, Ageing, Immunoglobulin isotypes

## Abstract

**Background:**

The immune system undergoes several alterations of innate and adaptive immunity during ageing. The main features of the aged immune system are a reduced diversity of T cell receptors and a reduced activity of innate immune cells with subsequent changes in adaptive immunity resulting in a less effective, less specific, and dys-regulated immune response and in an increased susceptibility towards infection, malignancy, and autoimmunity. The process is referred to as immunosenescence and is also modulated by environmental modifiers, such as dietary factors. High fat diet (HFD), via direct modulation of immune cell function by fatty acids and/or increased body fat mass, influences immune function. However, it is not clear whether HFD is beneficial or detrimental for the functioning of the ageing immune system.

**Methods:**

Male Wistar rats fed with either a high fat diet (HFD 43 en% of fat) or control diet (SD, 25 en% of fat) over up to 24 month and were analyzed for plasma IL-1β, IL-6, TNF, IgM, IgG1, IgA, IgG2a, IgG2b, IgG2c, light chains lambda and kappa, testosterone, prolactin and percentage of splenic B cells and apoptosis rate, respectively.

**Results:**

In general, all analyzed immunoglobuline isotypes increased with age, except for IgA. This increase was attenuated by HFD. In HFD and SD rats the percentage of B cells in the spleen and also their apoptotic rate was lower in aged as compared to young animals with no additional diet-induced effect. Testosterone and prolactin levels were lower in old animals, as expected. There was a statistical trend towards an increased prolactin/testosterone ratio in middle aged (6–12 monthsnth) HFD rats as compared to SD. IL-6 was neither affected by HFD nor age. On the other hand, HFD rats showed a decrease in IL-1β as compared to SD, which correlated with the above-mentioned suppressive effect on immunoglobulin isotypes, especially IgM.

**Conclusion:**

In Wistar rats, HFD reveals an immunosuppressive effect in ageing animals by decreasing immunoglobulins, especially IgM, and IL-1β when compared to SD.

## Background

The term i*mmunosenescence* comprises functional, qualitative and quantitative changes in the immune system during natural ageing. These changes due to immunosenescence affect both the innate and the adaptive immune response [1]. In general the immune system of the aged individuals appears to be characterized by a preponderance of anti-inflammatory mechanisms, e.g., more IL-10 production by macrophages and a loss of the flexibility of the immune response towards new antigens mainly due to a retraction of the T cell receptor repertoire [2]. The ageing immune system relays more on memory responses and is less prone to effective adaptive responses to newly encountered antigens [2]. This results in a weaker response to vaccination, an increase in the risk of infection, and a less stringent surveillance against developing tumors [3].

The reasons behind the phenomenon of immunosenescence have not been fully elucidated yet. Moreover, there are several confounders such as body fat mass that influences the immune response independently, but might vary substantially within the heterogenous population of old people. It has been suggested that a high body fat mass does not have the same negative impact in the old as it has for a young population [4, 5]. This was concluded by studies that showed a survival advantage in a geriatric population above age 65 when body mass index was greater than 25 kg/m^2^, which is usually defined as overweight [6]. Therefore, the question whether a high body fat mass in aged persons has to be regarded as a beneficial rather than a harming factor is still a matter of debate. Notably, fat tissue and embedded innate immune cells are known to produce mediators that directly affect the immune function, such as adipokines or several - mostly proinflammatory – cytokines [7], which might have a positive effect in old people because their immune system is *per se* in a more suppressed state [2]. On the other hand, fatty acids *per se* have immunomodulatory effects [8], which raises the difficulty of separating effects of a high fat diet (HFD) resulting from different diet composition, from effects caused by increased body fat mass, or merely increased calorie intake. It follows that the balance of cytokines, given by proinflammatory (such as IL-1β, IL-6, TNF) and anti-inflammatory effector molecules (such as IL-10) depends on both ageing and obesity-related dietary patterns [9, 10]. The latter might superimpose immunosenescence either in a synergistic or antagonistic manner [11].

On this background and due to the known decrease in vaccination success and increased infection rates in aged people, we wanted to investigate whether a HFD would alter pivotal immune mediators and B cell function. We employed an earlier described rat model [12], where rats are kept on a isocaloric regimen to discern between effects due to different diet composition and increased calorie intake.

## Results

### Weight gain and survival rate

**Fig. 1:**
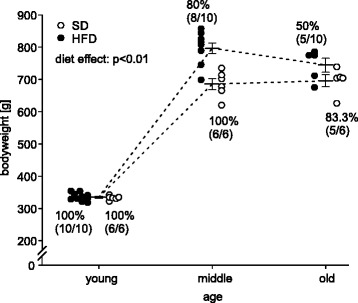
Weight course. Male Wistar rats were fed ad libitum with either a diet containing 25 en% (SD, open circle) or 45 en% (HFD, closed circle) of neutral fat. The diet was switched from ad libitum to a weight sustaining regimen from the age of 12 months onwards. Each animal is represented by one dot. Survival rates are given as percentage (number of animals at respective timepoint/number of young animals). Long solid line represents the group mean. Error bars represent the S.E.M. ANOVA for repeated measurement was used to determine differences between groups. Young: 2 month, middle aged: 6–12 month, old: 18–24 month

As expected there was an overall weight gain with age, and rats on HFD showed increased body weight in middle aged rats as compared to rats on standard diet (SD, Fig. 1). From month 12 onwards there was an isocaloric intake of approximately 0.12 kcal/g body weight per day [12], leading to a decrease of bodyweight difference between HFD and SD in the old age group as compared to middle aged rats. As also shown in Fig. 1, there was a difference in death rates between the two dietary regimens, with 83.3 % (*n* = 5) in the SD group and 50 % (*n* = 5) in the HFD group reaching the old age group (18–24 month).

### Immunoglobulin isotypes, especially IgM are suppressed by HFD in old rats

**Fig. 2:**
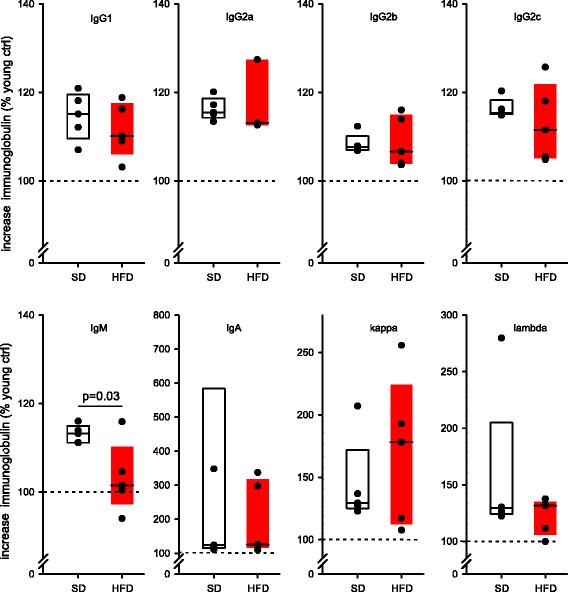
Relative change of plasma isotypes for each individual animal over time. For the longitudinal analysis over time, only animals reaching the old age group (HFD: 5, SD: 5) were considered. Values are based on intra-individual data-sets and expressed as a percentage change relative to the corresponding baseline in young rats (dashed line, 100 %). Each black circle represents the individual value of one animal. Data are presented as box plots (HFD: red box; SD: white box), the solid line representing the median of the group. ANOVA for repeated measures was used to determine differences between dietary regimens and changes over time. One graph represents one isotype as labelled. Young: 2 month, old: 18–24 month

To determine whether HFD and/or age influences basic B cell function, we determined several immunoglobulin isotypes and light chains in plasma of young and old rats, respectively (Fig. 2). The results are expressed as intra-individual percent change as related to levels in young animal, respectively. The analysis revealed an overall increase in all determined isotypes and light chains with age, but independent of diet (Age-effect: p-values_repeated measure ANOVA_: IgG1, IgG2a, IgG2b, IgG2c: *p* < 0.0001; IgM: *p* = 0.02; IgA: *p* = 0.07; kappa: *p* = 0.006; lambda: *p* = 0.03). However, when separating SD and HFD-rats in this analysis, the above-mentioned age-dependent increase of isotypes in the HFD group only remained significant for IgG2a (*p* = 0.03), indicating a general suppressive effect of HFD on immunoglobulin production. On the other hand, a significant decrease of immunoglobulin levels in HFD rats as compared to rats on SD was only detected for IgM in post-hoc analysis (*p* = 0.03, Fig. 2). Taken together, HFD appears to directly and/or indirectly inhibit B cell function resulting in decreased immunoglobulin production, especially IgM.

### Splenic B cells numbers and apoptosis are not affected by HFD but decline with age

**Fig. 3:**
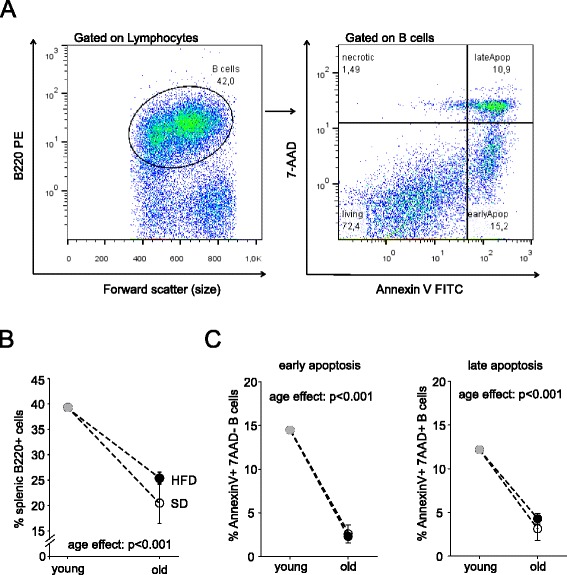
Effects of a high fat diet (HFD) vs. standard diet (SD) on percentage of splenic total and apoptotic B cells over time. **a** Density plots depicting gating strategy for B220+ cells in the spleen (left plot) and analysis of early (Annexin V+/7-AAD-) and late (Annexin V+/7-AAD+) apoptotic B cells in the pleen (right plot). **b**, **c** Circles represent mean values at different age groups [grey circle: baseline value, closed circles: HFD, open circles: SD]. **b** percentage of splenic B220+ B cells. **c** percentage of early apoptotic (left graph) or late apoptotic (right graph) B cells in the spleen. ANOVA was used to determine overall difference between dietary regimen and age groups. Error bars represent the S.E.M. Young: 2 month, old: 18–24 month

Since the observed changes in plasma immunoglobulin (Fig. 2) might be caused by an effect of HFD on B cell survival, percentages of total and apoptotic splenic B cells were determined in the HFD- and SD-group, both in young and aged animals (Fig. 3). The gating strategy is shown in Fig. 3a. The percentage of B220+ B cells in the spleen was not altered by HFD vs. SD. However, old rats showed clearly decreased levels as compared to young animals (Fig. 3b). Although it has been suggested that HFD increases B cell apoptosis [13], HFD did not alter percentage of early or late apoptotic B cells in our experiment (Fig. 3c). However there was a decrease of apoptotic B cells with age independent of diet (Fig. 3c).

### Age but not diet influences testosterone and prolactin plasma levels

**Fig. 4:**
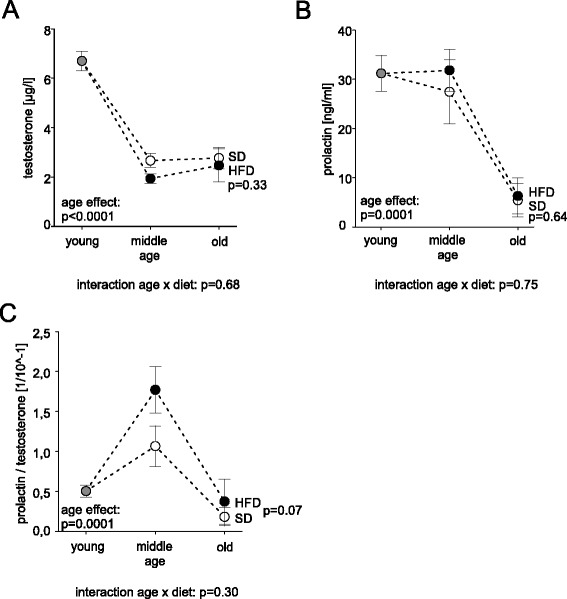
Level of testosterone and prolactin over time and between dietary regimens (HFD vs. SD). Circles represent mean values at different age groups [grey circle: baseline value, closed circles: HFD, open circles: SD]. **a** Plasma level of testosterone. **b** Plasma level of prolactin. **c** Prolactin to testosterone ratio over time. ANOVA was used to determine overall difference between dietary regimen, age groups, and possible interaction (AgeDiet). Error bars represent the S.E.M. Young: 2 month, middle aged: 6–12 month, old: 18–24 month

Hormones like testosterone and prolactin, respectively influence the function of immune cells [14, 15] and are correlated with age [16] and body weight [17, 18]. Therefore, serum levels of testosterone and prolactin were determined in plasma of young, middle-aged, and aged rats (Fig. 4). Testosterone levels were decreased in middle aged rats (6–12 mo) as compared to young animals and remained at this lower level in old rats (Fig. 4a). Prolactin was not decreased in middle aged rats as compared to young animals, however, in old rats levels were lower as compared to middle aged and young animals (Fig. 4b). For both hormones no difference was detected between HFD and SD (Fig. 4a, b). Since the level of the anti-inflammatory testosterone dropped before the levels of proinflammatory prolactin, we were interested in the ratio of both hormones. The prolactin/testosterone ratio increased in middle aged rats in favor of the proinflammatory prolactin (Fig. 4c). There was a statistical trend towards an increase in the prolactin/testosterone ratio in HFD as compared to SD rats (*p* = 0.07, Fig. 4c). Taken together, neither testosterone nor prolactin was influenced by HFD in our model. However, the kinetic of changes for the two hormones in the process of ageing might be influenced, resulting in an increased prolactin/testosterone ratio in midlife of male rats.

### HFD affects IL-1β but not IL-6

**Fig. 5:**
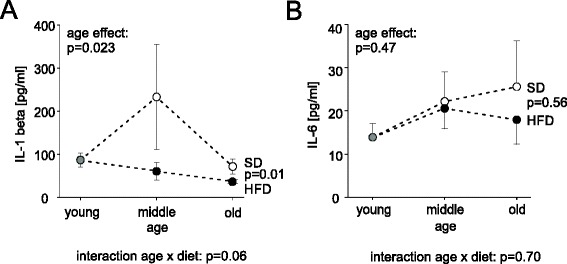
Amount of cytokines IL-1β and IL-6 over time and with different dietary regimens. Circles represent mean values at different age groups [grey circle: baseline value, closed circles: HFD, open circles: SD]. **a** Plasma level of IL-1β. **b** Plasma level of IL-6. ANOVA was used to determine overall difference between dietary regimen, age groups, and possible interaction (AgeDiet). Error bars represent the S.E.M. Young: 2 month, middle aged: 6–12 month, old: 18–24 month

It has been shown that mediators like IL1-β, TNF, and IL-6 depend on body weight and age, are influenced by the dietary regimen, and might affect B cell function [14, 19, 20]. Therefore, levels of IL1-β, TNF, and IL-6 were determined in plasma samples from young, middle-aged, and aged HFD and SD rats, respectively. As shown in Fig. 5a, IL-1β was suppressed from 6 month of age until the end of experiment at 24 month by HFD as compared to SD (diet effect: *p* = 0.01, Fig. 5a). Age was not a confounding factor for this HFD-induced suppression (interaction age × diet: *p* = 0.06, Fig. 5a). In contrast, there was no influence on IL-6 levels, neither by age nor by diet (Fig. 5b). As mentioned above, TNF was not present at high enough levels in plain plasma for valid detection with ELISA. Neither IL-1β nor IL-6 correlated with bodyweight (IL-1β: *r* = -0.073, *p* = 0.61; IL-6: *r* = 0.150, *p* = 0.295). Taken together, HFD leads to a differential effect on proinflammatory mediators with decreased IL-1β and no influence on IL-6.

## Discussion

The ageing immune system is challenged by numerous intrinsic changes in immune cell pool and function [21]. The activation of B cells, and their production of highly antigen-specific antibodies is the result of a complex interplay between antigen-presenting cells, antigen-specific T cells, and structural prerequisites. Therefore, determining B cell functionality by determining antibody isotype levels provides a general impression of adaptive immune system function. Physiological changes with age might be modulated by numerous external stimuli. Dietary factors are such modulators, and long term exposure to certain diets have been shown to modulate immune function in either a bad or a good way [11, 22–25]. Infectious diseases, less effective tumor surveillance, and an ineffective response to vaccination are resulting problems of immunosenescence in a geriatric population [26, 27]. In geriatric research there have been reports demonstrating a positive correlation between higher body mass and longevity [4–6]. Therefore, one possible hypothesis is that a sustained HFD increases body mass, which is beneficial in advanced age by augmenting immune system functionality, maybe by just providing enough energy for effective immune responses [28]. However, we could not substantiate this hypothesis. Our rats on HFD showed lower survival rates than rats on SD, which correlates with inhibition of B cell function in the HFD group as compared to SD, indicating immunosuppressive effects of HFD rather than the opposite. Of course, human data and our data in rodents are not directly comparable and we only studied the physiological response to HFD as compared to SD, and did not subject our animals to an immunological challenge, as is the case in a large human epidemiological analysis, where some immunological challenge always takes place. Planed studies in rodents applying an immune challenge will give further insight regarding this question.

Alteration in antibody isotypes with age are expected [29–32]. Results from human studies are mostly consistent with our findings and show a general, age-dependent tendency towards an increase in antibodies of different isotypes over time. However, in human studies not all measured isotypes are increased with age and there is a more specific effect on certain isotypes, e.g., IgA and/or IgG but not IgM. Since the youngest rats in our study with 1.5 months of age have mostly not been exposed to many foreign antigens, the basal immunoglobulin levels in these animals were minimal. In the human studies, the youngest participants were already at least 20 years of age, and supposedly have been subjected to more infectious agents and other antigens than our young experimental laboratory animals. Therefore, the baseline level of all isotypes measured is assumably already high in the human studies and an increase with age is harder to detect. In our study, the aged-dependent increase in all isotypes in SD fed rats with age might reflect the cumulative exposure to various antigens over time. Notably the age-dependent increase of immunoglobulins seems to be a rather unselective effect, as it affects all measured isotypes and light chains in more or less the same manner.

In the literature there are some hints that HFD might augment immunoglobulin levels. This notion is mainly based on the hypothesis that obesity *per se* increases inflammation, i.e., proinflammatory cytokines like IL-1β and IL-6 due to an increased leakiness of the gut barrier in obese individuals [33–36]. We did not find any correlation between IL-1β and/or IL-6 with body weight, respectively in our study sample. Moreover, IL-1β was even suppressed in rats fed HFD as compared to SD, whereas the diet had no influence on the plasma levels of IL-6. It has been also suggested that saturated fatty acids, which were present in our HFD, directly activate pattern recognition receptors, like Toll-like receptors, and subsequently increase IL-1β production by monocytes (e.g., [8]). On the contrary, another study demonstrated that HFD in rats did not alter production of cytokines by adipose tissue rather than alter cytokine production in the brain [37].

However, most of these studies showing a correlation between HFD, bodyweight, and proinflammatory effects do not distinguish between direct effects of HFD and indirect effects by developing adipositas or providing excessive calories. Our study is different in this respect, because excessive weight gain was not observed from 12 months onwards. We therefore propose a direct anti-inflammatory effect of HFD, with decreased levels of IL-1β and no difference in IL-6 when compared to concurrent control rats fed with SD. In this respect, anti-inflammatory effects of fatty acids are also known especially for unsaturated fatty acids, like oleate [38], which was part of our HFD regimen. Another possible explanation for the immunosuppressive effect of HFD vs. SD is the difference in diet composition. In HFD rats, needed calories are provided dominantly by fatty acids, whereas in SD, carbohydrates play a more important role. However, immune cells need a lot of energy when activated [28] and the exploitation of energy from carbohydrates is much easier than the use of fatty acids. Therefore, HFD rats might simply be hindered in activating immune cells, because energy is mainly provided by fatty acids, which are not as readily usable by immune cells than carbohydrates.

Taken together, HFD might evolve a direct anti-inflammatory potential, which has to be differentiated from indirect inflammatory effects mediated by obesity. This anti-inflammatory effect of HFD in terms of suppressing IL-1β was most distinctive in middle aged rats. Since IL-1β increases antibody production and proliferation at least in human B cells [39] the decreasing effect of HFD on immunoglobulin isotypes with a profound effect on IgM levels might be due to decreased IL-1β in HFD rats. A similar specific effect on IgM was observed in C57B6 mice when subjected to HFD [40]. In this latter study specific IgM but not IgG or IgE levels were decreased in HFD mice as compared to controls. Total blood cell count and differential were not altered in HFD mice [40], however, the number of B cells in the spleen was not determined. In another study using C57B6 mice, HFD-induced oxidative stress reduced B cell numbers in the spleen by increased apoptosis [13]. In the present study, the percentage of splenic total and apoptotic B cells declined with age, but diet showed no influence. The difference of the results could be due to differences in diet composition, since HFD in the above cited study was compared to a control diet with a very low fat content (4.9 % fat w/w), whereas our SD regimen contained (25 % fat w/w). Therefore, the two studies are not directly comparable. It is possible that our SD diet already induced apoptosis in B cells, which is not augmented by HFD any further.

It has been first described in 1999 [41] and since then shown in numerous studies that leptin is increased under the influence of HFD as compared to control diets in rats (e.g., [42–45]). It also has been shown in rats, using the same dietary regimen that HFD increases leptin levels [12]. Leptin can even be increased in rats by HFD within 72 h independent of an increase in bodyweight [42]. Leptin has not been determined for the current analysis, which is a limitation of our study. However, an increase in leptin would not explain immunosuppressive effects of HFD in our model, because leptin acts rather stimulatory, at least on human B cell [20].

We observed that rate of B cell apoptosis declined with age, a phenomenon termed age related apoptosis resistance and already described for aged fibroblasts, which are less sensitive to apoptosis inducing agents as compared to young fibroblasts, possibly due to reduced translocation of stress induced signaling molecules into the nucleus [46]. Our data also show decreased rates of apoptosis among B cells in the spleens of old as compared to young rats independent of diet. Therefore, a similar mechanism as described for fibroblasts might be in place for B cells, accounting for decreased apoptosis in aged rats independent of diet.

We also determined hormone levels of the known pro- and anti-inflammatory molecules prolactin and testosterone, respectively, since these hormones might also be influenced by HFD [47, 48] and have been shown to correlate with inflammatory parameters [49, 50]. However, we found no influence of HFD on levels of testosterone or prolactin. For testosterone an influence was expected, since it has been shown that HFD suppresses testosterone levels in male Wistar rats [47]. However, rats in this study were fed HFD from the beginning of life as opposed to starting at 8 weeks in our study and the control diet in this study had a lower fat percentage as compared to SD in our study (10 % vs. 25 %), possibly pronouncing HFD effects. Therefore, the two studies are not directly comparable. The trend towards an increased prolactin/testosterone level in HFD rats would support a proinflammatory milieu in middle aged rats. However, in aged rats this difference is abolished again. Therefore, this finding cannot be used to explain the decrease in IgM in old HFD rats. In contrast, at an increased prolactin/testosterone level, IgM levels are expected to increase [51, 52]. However, the stimulating effect of prolactin on immunoglobulin production seems to be only observed when B cells are activated, whereas in a resting state B cells seem to be less responsive to prolactin [53]. In the present study mainly resting B cells are targeted because no immunization protocol was used. Therefore, although prolactin/testosterone is increased, IgM levels are not affected in our setting.

## Conclusions

Taken together, our data support the hypothesis that HFD in old rats has immunosuppressive effects including reduced IL-1β and reduced immunoglobulin levels, especially IgM. Of course, these are correlative data and the causal relationship between the different players needs to be determined in future studies. Also, this is only a description of the basal state and immunization studies need to be performed to complete the picture. However, our data show that HFD on its own, independent of bodyweight and caloric intake shows immunosuppressive effects that are evident in old rats.

## Methods

### Experimental animals

Male Wistar rats were purchased at the age of 8 weeks from Charles River (Sulzfeld - Germany). The rats were caged in groups of 4 with free access to water and to different dietary regimes as described below. Animals were held on a 12:12-h light–dark cycle. Rats were allowed to accommodate to the new surroundings for at least one week before data collection. All procedures were approved by the local animal rights committee and complied with the German Law on Animal Protection as well as the UFAW Handbook on the Care and Management of Laboratory Animals, 2010.

### Dietary regimen

Rats were fed with a HFD or a SD as described before [12]. Briefely, for the longitudinal study 16 rats were fed *ad libitum* with a rodent chow in pellet form containing either 25 en% (standard diet, SD) or 43 en% (high-fat diet, HFD) of fat (Altromin GmbH, Lage - Germany). For analysis of B cell numbers and apoptosis rates in the spleen, five additional animals have been analysed in the young age group. The fat component in both diets was based on a constant mixture of lard and corn oil corresponding to a molar ratio of 1.2 : 1 between long-chain saturated fatty acids (mainly palmitate 16:0, stearate 18:0) and long-chain monounsaturated fatty acids (mainly oleate 18:1). The difference in the quantity of fat between SD and HFD was compensated for by a different content of complex carbohydrates (53 en%, SD versus 37 en%, HFD), while the protein content (22 en%, SD versus 20 en%, HFD) as well as the amount of essential micronutrients were equal in both diets.

### Blood samples

Blood samples were drawn after an overnight fast (16 h) either from the tail vein or from the heart of the sacrificed animals at indicated time-points and transferred into EDTA-coated vials. Plasma was immediately prepared and stored at -20 °C pending further analysis. Prolactin and testosterone were measured by routine procedures (ADVIA Centaur, Siemens, Erlangen, Germany). Insulin, IL-1β, TNF, and IL-6 were measured using rat-specific ELISA kits (Mercodia, Uppsala - Sweden; Linco Research, St. Charles - U.S.A., Millipore, Billerica - U.S.A.).

### Immunglobulin isotyping

Amounts of isotypes IgA, IgM, IgG1, IgG2a, IgG2b, IgG2c, and light chains lambda and kappa in plasma were determined by commercially available ELISA following the manufacturer’s instructions (rat immunoglobulin isotyping ELISA kit, BD Pharmingen). Briefly, 96-well plates were coated over night at 4 °C with mouse anti-rat of the specific isotype, respectively. After blocking and incubation of the samples, plates were washed and bound isotypes were detected by HRP-labelled mouse anti-rat immunoglobulin detection antibody. After washing and adding colour reagent plates were read at 450 nm. O.D., values at 570 nm were subtracted. O.D. values were standardized to the respective positive control on the respective plate to avoid inter-assay differences. According to the manufacturer’s technical data sheet, the anti-rat IgG2a antibody pair might cross-react with IgG1 in some instances. To estimate the degree of possible cross-reaction, we correlated results for the two isotypes IgG2a and IgG1 in all available samples measured and did not find any correlation between IgG1 and IgG2a levels in our dataset (*r* = 0.259, *p* = 0.271). Therefore, we assume that the possible cross-reaction of anti-IgG2a with IgG1 plays a negligible role in our specific setting.

### FACS analysis

For analysis of the percentage of splenic B cells and apoptotic B cells, spleens were removed from animals at indicated time points. Single cell suspensions were prepared by passing spleens through a nylon mesh, followed by red cell lysis. 1×10^6^ splenocytes were stained with PE-labelled mouse anti-rat B220 antibody (AbD Serotec, clone OX-33), FITC AnnexinV (BD Bioscience), and respective isotype control antibody. After washing the cells, FACS analysis was performed on a FACS Calibur (Becton Dickinson Immunocytometry Systems, San Jose, CA). All flow cytometry data were analyzed with FlowJo Software (V.7.2.4., Tree Star Inc., Oregon, USA).

### Statistical methods

ANOVA was used to determine overall effects of age and diet. Subsequent post-hoc tests were used to compare the effect of HFD to SD at selected time points. Data of different time points were grouped into three categories, young (including data 2-3 month), middle aged (including data 12–18 month), and old (including data 18–24 month). All analyses were conducted using IBM SPSS Statistics for Windows (International business machines cooperation, Version 20.0.0.1). p-values less than 0.05 were considered significant.
